# Raptors bred in captivity: semen characteristics and assisted reproduction outcome in goshawk (*Accipiter gentilis*)

**DOI:** 10.7717/peerj.15094

**Published:** 2023-03-22

**Authors:** Anna Maria Fausto, Anna Rita Taddei, Federica Batocco, Maria Cristina Belardinelli, Marcella Carcupino, Achille Schiavone, Sergio Saia, Annelisse Castillo, Margherita Marzoni

**Affiliations:** 1 Department for Innovation in Biological, Agrofood and Forest systems, University of Tuscia, Viterbo, Italy; 2 Center of Large Equipments, Section of Electron Microscopy, University of Tuscia, Viterbo, Italy; 3 Department of Veterinary Medicine, University of Sassari, Sassari, Italy; 4 Department of Veterinary Sciences, University of Torino, Grugliasco, Italy; 5 Department of Veterinary Sciences, University of Pisa, Pisa, Italy

**Keywords:** Goshawk, Captive breeding, Raptor species conservation, Season, Semen characteristics, Sperm ultrastructure

## Abstract

Three sexually mature goshawks reared in captivity and imprinted on humans to express reproductive behavior according to the cooperative method were studied for three consecutive breeding seasons to assess the quality of their sperm. The following parameters were analyzed: ejaculate volume and sperm concentration, motility, viability, and morphology. Ejaculate volume, sperm concentration and motility fluctuated along the reproductive season, revealing the greatest quality of the reproductive material at full springtime (*i.e*., April). Motility of the sperm collected in March strongly reduced with age, contrary to samples collected in April or May. Sperm viability was not influenced by either age or month of collection within each season. Ultrastructural investigations provided information on normal sperm morphology for the first time in this species. The morphological categories of sperm defects in fresh semen, present at low percentages, are also described. Functional analyses (perivitelline membrane assay and artificial inseminations) confirmed the good quality of the semen obtained using the cooperative method. The reported data provide the basis for further studies aimed at developing protocols to improve the outcome of artificial insemination and semen cryopreservation in the goshawk as well as other bird of prey species.

## Introduction

The ongoing worldwide decline in raptor populations is well documented. The main causes have been ascribed to the fragmentation and loss of habitat, environmental diseases, lack of prey, and hunting (IUCN, 2021). Of the various proposals put forward aimed at promoting bird species preservation, one of the most important concerns *ex situ* conservation, based on the reproduction and propagation of individuals in captivity (Blanco et al., 2002; Saint Jalme, 2002; Blanco, Bird & Samour, 2007; Bailey & Lierz, 2017; Martin-Wintle et al., 2019).

In captivity, birds of prey are either allowed to mate naturally or are subjected to assisted reproduction (Bailey & Lierz, 2017). The latter offers two key advantages: it can help increase genetic diversity by using under-represented males (Waldoch et al., 2007; Blanco et al., 2009) and boost the remaining populations by returning captively bred birds back into the wild (Neumann, Kaleta & Lierz, 2013; Dogliero et al., 2016). Considering this context, reproductive biotechnologies, such as artificial insemination (AI) and semen cryopreservation, have acquired an increasingly important role in wild bird species preservation, and their application is receiving research attention (Samour, 2004; Madeddu et al., 2009; Schneider et al., 2019; Cardoso et al., 2020; Piacek et al., 2020).

AI increases fertility rates and the number of offspring obtained from specific individuals. It improves genetic exchange between populations without the need for transferring live animals, and it has helped establish gene banks in the form of frozen semen (Łukaszewicz, Kowalczyk & Rzońca, 2015; Villaverde-Morcillo et al., 2015).

One of the most important traits determining fertility success is the quality of the collected semen, which depends, among other things, on the semen collection procedure (Castillo et al., 2021). The difficulty in obtaining good quality sperm from animals lies in an internal conflict which usually affects wild and captive-bred birds when they are handled (Łukaszewicz, Kowalczyk & Rzońca, 2015). Indeed, wild animals bred in captivity are well known for their typically nervous disposition, and the handling of animals during sperm collection procedures causes additional stress to the animals, affecting the quality of the semen itself and often making it unusable for its intended uses, that being artificial insemination and cryopreservation (Madeddu et al., 2009). In particular, raptors kept in captivity exhibit difficulties in expressing normal sexual behaviors, with aggression often manifesting between partners during courtship (Gee et al., 2004; Dogliero et al., 2016). The use of the cooperative breeding method offers obvious advantages as it involves the use of imprinted males which are quieter in their temperament towards the breeder. In this particular type of breeding, the males, isolated from conspecifics, socialize and express sexual behaviors towards the human, releasing their sperm spontaneously (Temple, 1972; Gee et al., 2004; Villaverde-Morcillo et al., 2015). This semen collection technique has been used with high success in a number of birds of prey species, such as *Aquila chrysaetos* (Grier, 1973; Villaverde-Morcillo et al., 2015), *Buteo jamaicensis* (Temple, 1972) and *Falco mexicanus* (Bailey & Lierz, 2017).

Successful AI programs require knowledge about specific semen features in order to evaluate the quality of both the material collected as well as the donor itself, selected by humans with the specific aim of passing his attributes on to the progeny. Indeed, semen evaluation is of the utmost importance for assessing male fertility and for judging semen suitability for the cryo-preservation process (Łukaszewicz & Kowalczyk, 2015; Al-Daraji & Al-Shemmary, 2016; Santiago-Moreno et al., 2016; Cardoso et al., 2020; Castillo et al., 2022).

The semen characteristics of a number of bird of prey species bred in captivity were recently described with the scope of improving breeding techniques (Dogliero et al., 2016; Frediani et al., 2019; Fischer et al., 2020; Piacek et al., 2020). However, much more research is needed to obtain semen quality data that will be useful for testing appropriate techniques on rare avian species and for developing reliable, applicable protocols for cryopreservation. The examination of quantitative and qualitative variables, such as semen volume, sperm concentration, sperm motility, sperm viability and morphological abnormalities, can provide a valuable way to predict fertilization potential (Santiago-Moreno et al., 2009).

Furthermore, although morphology, together with sperm motility and sperm concentration, has been identified as one of the most important semen quality parameters, little information is available in the literature on raptor semen traits (Madeddu et al., 2009) or raptor sperm fine structure (Santiago-Moreno et al., 2009; du Plessis & Soley, 2011, 2014; Jamieson, 2011).

As regards the goshawk (*Accipiter gentilis*—Linnaeus 1758), only limited information is available on male reproductive aspects. Some data is available on the quality of manually collected semen, but for only a short interval of the reproductive period (Piacek et al., 2020), whereas no information has been published on the use of other collection methods or on whether the performances of males chosen as sperm donors remain high in subsequent reproductive seasons. Furthermore, almost nothing is known about the morphology and ultrastructure of the goshawk spermatozoon. The aim of the present article was to fill the gap in the literature by providing data on the semen characteristics of *A. gentilis* and assisted reproduction outcomes. Although this species is not currently considered at risk of extinction, it is known to be highly sensitive to habitat destruction, air pollution and unfavorable forestry management; thus, it could certainly become an at-risk species given the speed with which the natural habitats in Europe are changing (IUCN, 2021).

In the present work, three male and three female goshawks at full sexual maturity were reared in captivity and imprinted on humans to express reproductive behavior according to the cooperative method. They were studied for three consecutive breeding seasons in order to verify the quality of the sperm obtained with the cooperative copulation.

In particular, the quality of the semen produced by each individual during the three reproductive seasons was evaluated by analyzing the following parameters: ejaculate volume and sperm concentration, sperm motility and vitality, and the percentage of cytomorphological anomalies in the semen produced. In addition, the morphology and ultrastructure of the goshawk spermatozoon were described for the first time by both scanning (SEM) and transmission (TEM) electron microscopy.

Finally, functional analyses were performed *in vitro* by means of perivitelline membrane assay, using membranes obtained from laid heterologous eggs (chicken eggs), and *in vivo* by artificial inseminations performed in egg-laying female goshawks, in order to examine the fertilization potential of semen given by the cooperative method.

The present study aims to achieve a better understanding of the semen characteristics of the goshawk in order to optimize captive breeding techniques and AI methods for this species. Additionally, our results provide some insight into sperm donor choice and the role of seasonality in AI success in raptor species.

## Materials and Methods

### Goshawk maintenance

The goshawks (*A. gentilis*) used in this study were kept at the Breeding Centre for Birds of Prey named “European falcons” (FOI-RNA: 03DN) in Assisi (Perugia, Italy). This study was approved by the Ethics Committee of Pisa University (Ref.: OPBA_25/2021) under article 2, paragraph 1, letter b of the Italian Legislative Decree n. 26/2014.

Six unrelated animals, three males and three females, were used in the study. Males were evaluated during the reproductive seasons, from March to May, of three consecutive years. The animals were aged 5, 6 and 7 years old at the beginning of the experimental period. The body weight of the male birds ranged from 840 to 1,050 g. Three female goshawks were used for the *in vivo* evaluation of semen in the third year of study. The females were aged 7, 8 and 10 years old, and their body weight ranged from 1,050 to 1,480 g.

Each bird was kept individually in 2 m × 2 m × 3 m (w × l × h) aviaries, and males and females were not within visual range of each other. Animals were exposed to a natural photoperiod, and water was offered *ad libitum*. The weekly diet consisted of commercial quails, day-old chicks and pigeons (in a 5:1:1 ratio), plus vitamin and mineral supplementation. Food was offered once a day by the same operator. At the beginning of the breeding season, a wicker basket was added to each aviary housing females to allow them to build a nest.

### Semen collection

During the three reproductive seasons, males were stimulated to release semen twice a week by the same operator using the cooperative method; from 16 to19, from 20 to 24 and from 14 to 20 valid ejaculates in March, April and May, respectively, were collected for analyses. In accordance with the literature (Samour, 2004), the operator stimulated the animals by means of different calls, presented them food and mimicked their gestures. During the reproductive season, the human-bird interactions were intensified, and once the necessary level of conditioning had been achieved birds were enticed to copulate on the gloved hand of the operator; in this way, the male birds voluntarily performed the same maneuver that they would with female partners under natural conditions. The ejaculate of each goshawk was individually collected in 75 µL capillary tubes, and the samples were used for artificial insemination and qualitative analysis.

The capillary tubes with semen destined for qualitative analysis were maintained at 4 °C during the transport to the laboratory within 2 h from collection, whereas ejaculates destined for use in AI were immediately used at the European Falcon Centre.

### Evaluation of semen characteristics

The ejaculate of each animal was evaluated for volume, sperm concentration, sperm motility and the rate of live and normal sperm. The ejaculate volume was measured by aspirating the semen into a calibrated positive-displacement pipette. The sperm concentration was evaluated using the Makler chamber (AB Analitica, Padua, Italy) (Makler, 1980). In brief, spermatozoa were observed under a light microscope (40x) Zeiss (Axiophot) and the number of spermatozoa counted in a row of 10 squares indicated the sperm concentration in millions per mL unit.

Evaluations of sperm motility were made by light microscope using the Makler chamber filled with 10 µL of each sample to evaluate the number of motile cells in a total of 100 spermatozoa.

Sperm viability was evaluated using eosin-nigrosin staining (Bakst, 2010): the number of live and dead spermatozoa was evaluated by counting two sets of 300 sperm under a Zeiss (Axiophot) light microscope (40x) equipped with a color video camera (Axio Cam MRC), using a computer-assisted image analysis system (AxioVision).

The sperm penetration ability was evaluated *in vitro* by means of the sperm-egg interaction assay (Robertson & Wishart, 2010). The assay was carried out during the third reproductive season in March, April and May for a total of six times (twice each month), using laid chicken eggs due to the lack of goshawk eggs available for this purpose. Spermatozoa from the three males were incubated with sections of inner perivitelline layer (IPL) isolated from the same chicken egg as follows. The egg yolk was placed in a small dish and washed several times with 0.9% NaCl to remove excess egg white, and the IPL was then collected using fine tweezers. In a Petri dish, the IPL was washed several times with 0.9% NaCl to completely remove the yolk, then placed in 1 mL Dulbecco’s medium (Sigma Aldrich). A total of 5 μl semen was diluted 1:2 in pre-freezing Lake diluent (Lake & Ravie, 1981), added to the IPL (0.5 cm × 0.5 cm) and incubated for 5 min at 41 °C. A sperm suspension ranging from 1.5–2 × 10^7^ sperm/mL was used. Thereafter, membranes were cleaned in 0.9% NaCl, then transferred onto a glass slide to count under a light microscope the number of holes per unit area (1 mm^2^) produced by the sperm cells that penetrated the IPL. The whole procedure was done on plates heated to 41 °C.

### Ultrastructure of goshawk sperm

Samples were prepared for both scanning (SEM) and transmission electron microscopy (TEM) as previously described in Castillo et al. (2022). Specifically, all reagents and media were purchased by Electron Microscopy Science, Assing, Italy. For quantitative analysis of normal and abnormal sperm the specimens were observed under a JEOL JSM-5200 scanning electron microscope, considering 100 sperm cells from each goshawk from semen samples collected during the third reproductive season. Sperm abnormalities were classified according to du Plessis & Soley (2011). For detailed ultrastructure analysis of normal and abnormal sperm the specimens were examined under in a JEOL 1200 EX II transmission electron microscope and the image acquisition was performed by the Olympus SIS VELETA CCD camera equipped with iTEM software.

### Artificial insemination and offspring breeding

AI was performed in April using the cooperative method in females during the third reproductive season. According to the routine breeding practices at the European Falcon Centre, animals were combined as follows: the oldest female inseminated with semen from the oldest male (*i.e.*, oldest combination) through to the youngest female inseminated with semen from the youngest male (*i.e.*, youngest combination). AI was performed using a syringe connected *via* a tube to the capillary tube containing the pure semen. The females were stimulated by cloacal massage to facilitate the everting of the cloaca. Eggs were laid about 72 h after insemination, and further AIs were performed 2 to 6 h after each egg-deposition. Natural brooding was allowed and on the 15th day of incubation eggs were candled to establish successful fertilization and ascertain the stage of embryo development.

All goshawk chicks were reared by the natural mother until 8 weeks of age. They were weighed at hatch and on days 10 and 30. Their diet consisted of plucked and finely chopped pigeon and quail until day 10, small pieces of pigeon and quail until day 30, then whole prey thereafter according to the eating habits of this species.

### Statistical analysis

The experimental design was unbalanced in the replicates and consisted of two factors: age of the goshawks (referred as ‘A’, expressed in years, from 5 to 9 years old) and month of sampling (referred as ‘M’). The analyses were conducted along three consecutive years on individuals of, respectively, 5, 6, and 7 years old at the beginning of the study. Thus, month of sampling within years and within age of the individual, and years and age of the individual were, at the one time, replicated sources of variations. To overcome these issues, statistical analyses was conducted through a generalized linear mixed model (Glimmix procedure in SAS/STAT 9.; SAS Institute Inc., Cary, NC, USA). The model used was built for unbalanced designs (see Suardi et al., 2020; Bergonzoli et al., 2020 for details on procedure). Fixed factors were Age and Month nested within the Age (hereafter expressed as ‘M(A)’ or ‘Month (Age)’); random factors included Year of sampling. Age and Year were tested applying an autoregressive variance structure of the first order’ on the individual as a replicated unit in order to take into account of these factors as time-dependent factors. Unbiased estimates of variance and covariance parameters were assessed through a restricted maximum likelihood (REML) statement; and error degrees of freedom by the Kenward–Roger approximation. This correction takes into account that covariance parameter equal to zero does not contribute to the model, factors, and their interactions degrees of freedom. In order to separate means of factors and interactions, least square means (LSmeans) were computed and their *p*-differences compared by a Tukey-Kramer (TK) grouping at the 5% probability level. This allowed to carefully take into account the unbalancing of the experiment in term of individuals per age. Please note that the TK correction also adjusts the denominator degrees of freedom of each error term when multiple comparisons occur.

Lastly, LSmeans and their standard error estimates (SEE) per the interaction and single factors were provided in the Supplemental Materials along with the raw results and the syntax of the model applied.

## Results

### Semen collection and characteristics

Semen samples were collected through the cooperative method. Over the course of the 3 years considered, a total of 216 attempts were made, and valid ejaculates were obtained on 172 occasions (success rate: 80%).

The highest bird response rate to the operator’s calls occurred in April, during which, according to the breeder’s information, 90% of semen collection attempts were successful and provided uncontaminated ejaculates.

We evaluated 172 samples of goshawk semen. The ejaculate volume, sperm concentration, sperm motility, and sperm viability were 49.1 ± 23.8 µL, 41.0 ± 25.4 × 10^6^ cells/mL, 78.5 ± 10.3% and 85.3 ± 6.2% (means ± standard deviations), respectively.

**Figure 1:**
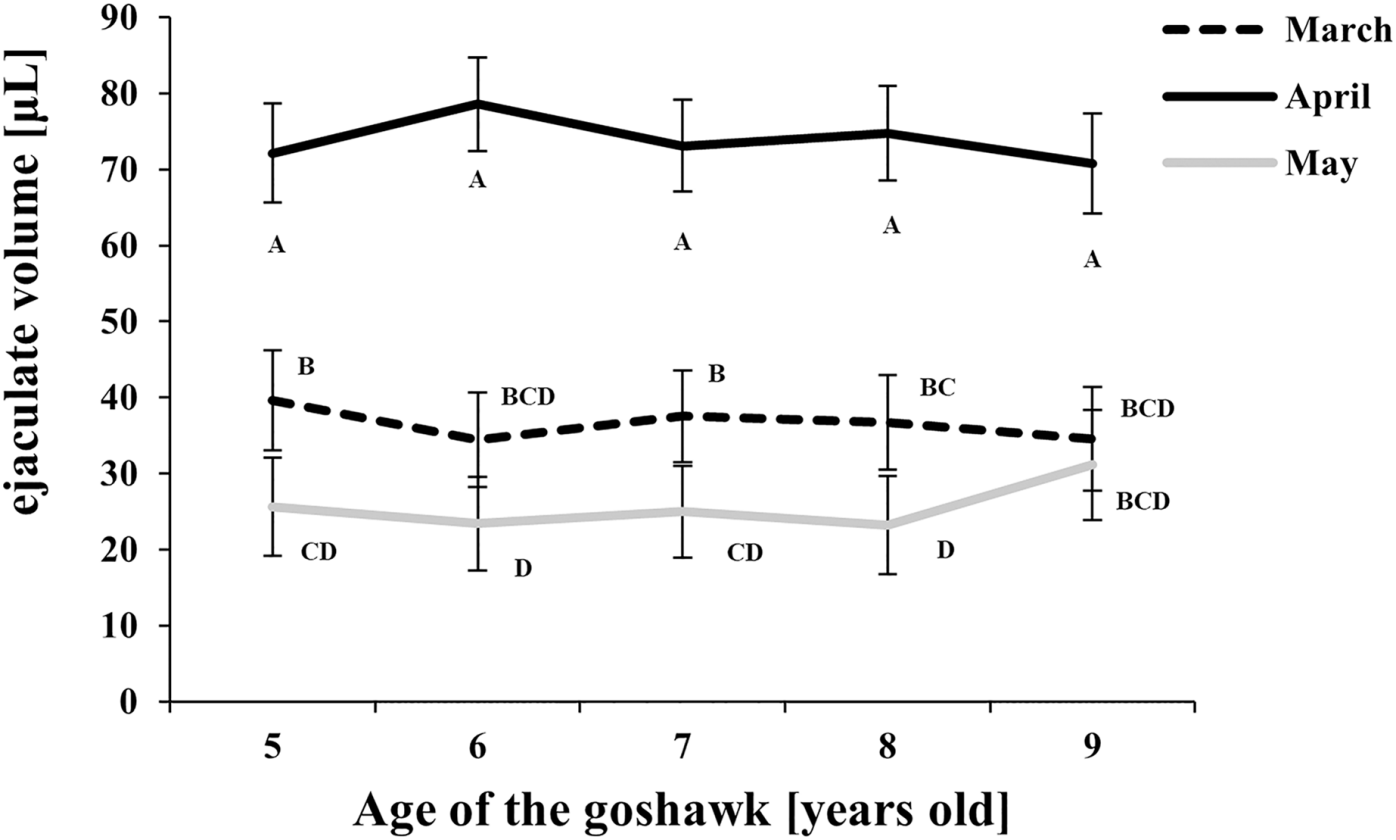
Effect of goshawk age (A, in years) and reproductive season month (M) on ejaculate volumes (LSmean ± Standard Error Estimates of the LSmeans (SEE)). LSmeans identified by identical letters were not considered significantly different according to Tukey-Kramer conservative (when applicable) grouping at the 5%. The grouping procedure did not reflect all significant comparisons. The following additional pairs appeared significantly different according to the *p*-difference: M3A6 *vs*. M5A6. See Supplemental Materials for the raw results of the statistical analysis and the arithmetic means and computed standard errors.

**Figure 2:**
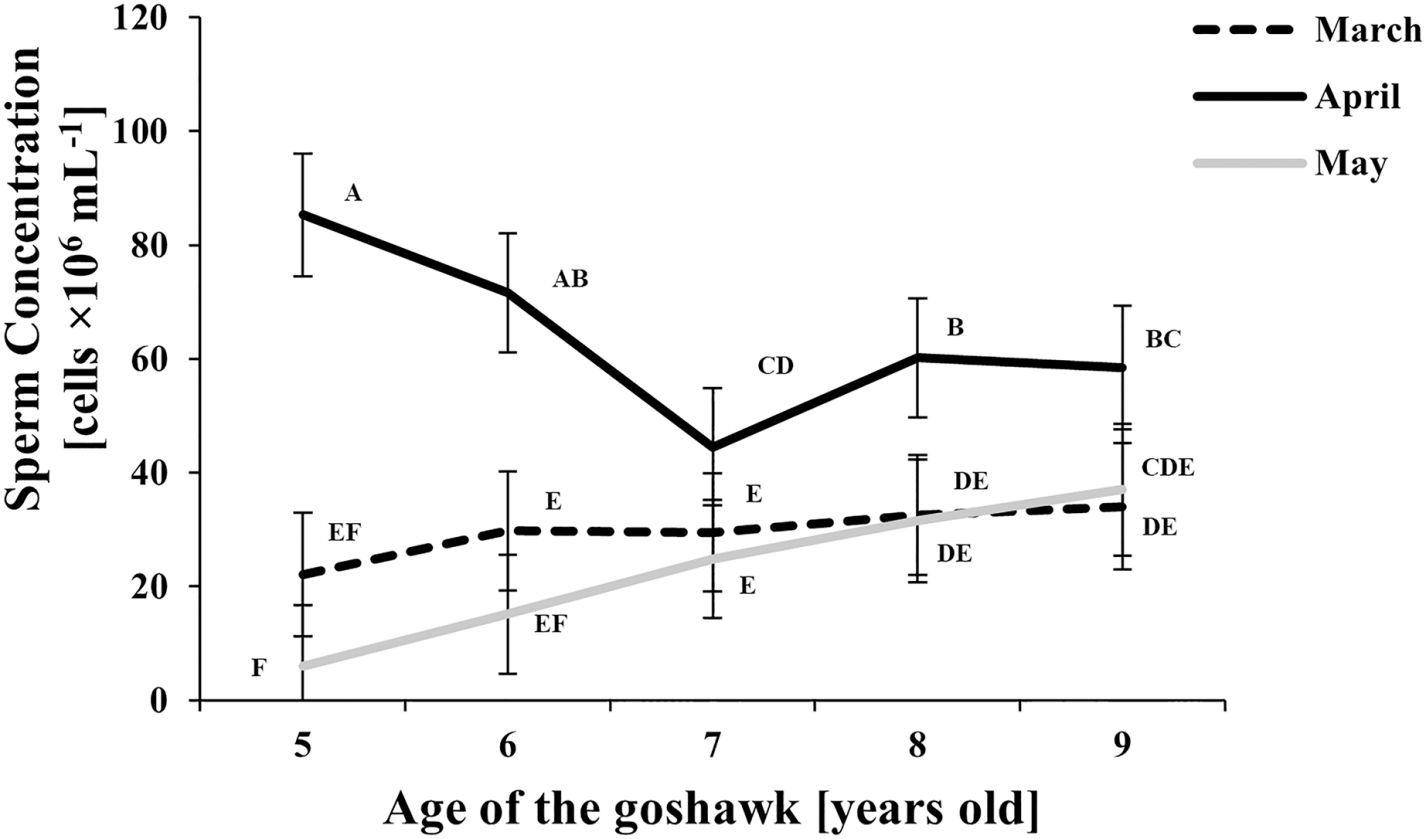
Effect of goshawk age (A, in years) and reproductive season month (M) on sperm concentrations (LSmean ± Standard Error Estimates of the LSmeans (SEE)). LSmeans identified by identical letters were not considered significantly different according to Tukey-Kramer conservative (when applicable) grouping at the 5%.The grouping procedure did not reflect all significant comparisons. The following additional pairs appeared significantly different according to the *p*-difference: M4A7 *vs*. M3A8; M3A9 *vs*. M5A6; M3A8 *vs*. M5A6; M5A8 *vs*. M5A6; M3A6 *vs*. M5A6; M3A7 *vs*. M5A6. See Supplemental Materials for the raw results of the statistical analysis and the arithmetic means and computed standard errors.

**Figure 3:**
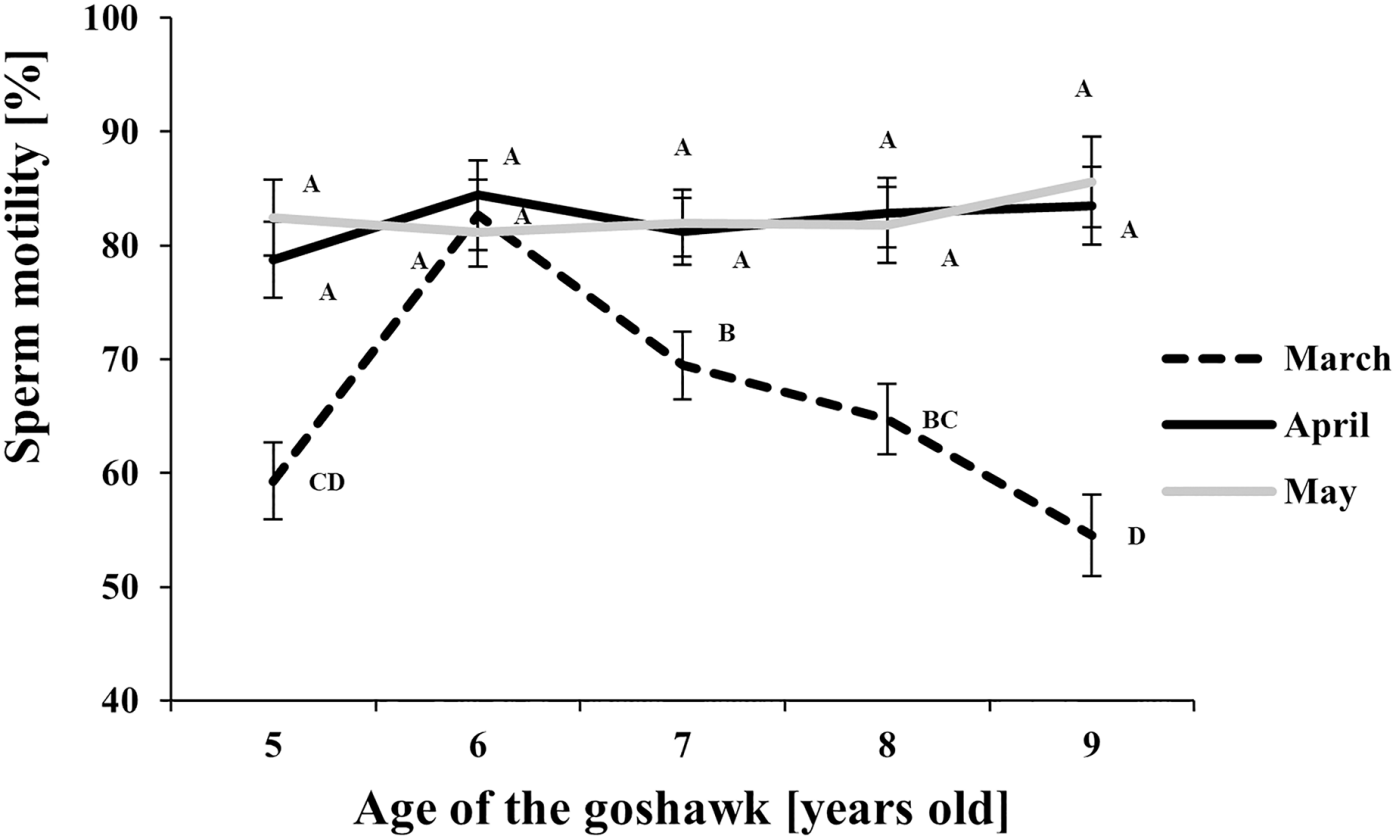
Effect of goshawk age (A, in years) and reproductive season month (M) on sperm motility (LSmean ± Standard Error Estimates of the LSmeans (SEE)). LSmeans with a letter in common should not be considered different according to Tukey-Kramer conservative (when applicable) grouping at the 5%. The grouping procedure does not reflect all significant comparisons. See Supplemental Materials for the raw results of the statistical analysis and the arithmetic means and computed standard errors.

**Figure 4:**
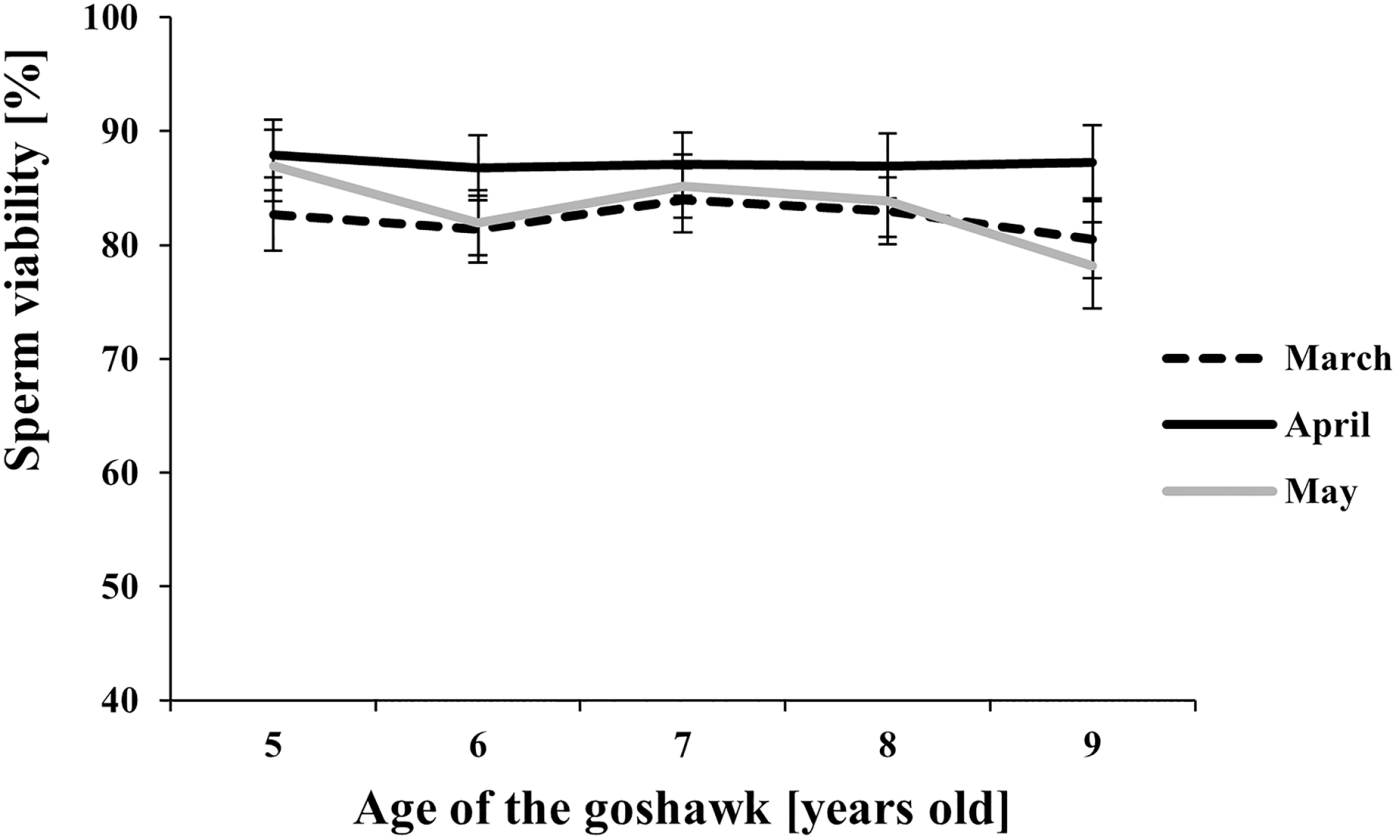
Effect of goshawk age (A, in years) and reproductive season month (M) on sperm viability (LSmean ± Standard Error Estimates of the LSmeans (SEE)). Tukey-Kramer conservative (when applicable) grouping at the 5% did not retrieve differences among treatments despite the M(A) interaction term showed a *p* = 0.0002. Unadjusted *p*-differences can be found in the Supplemental Material table “SAS results” from line 1,028 to line 1,135. See Supplemental Material for the raw results of the statistical analysis and the arithmetic means and computed standard errors.

The effect of age and month nested within age on semen traits is shown in Table 1 and details of the Month (Age) factor are shown in Figs. 1–4, where values reported are LSmeans and their standard error estimations.

**Table 1:** Type III tests of fixed effects for the variable studied. Probability < 0.05

Response variable	Effect	Numerator DF*	Denominator DF	F value	Pr > F
ejaculate volume (μL)	Age	4	6.6	0.04	0.9968
	Month (Age)	10	153.4	167.26	<0.0001
Sperm concentration (cells ×10^6^/mL)	Age	4	155.4	5.56	0.0003
	Month (Age)	10	155.0	74.78	<0.0001
Sperm motility (%)	Age	4	156.1	15.23	<0.0001
	Month (Age)	10	155.0	38.66	<0.0001
Sperm viability (%)	Age	4	156.1	2.43	0.0503
	Month (Age)	10	155.0	3.74	0.0002

Note:

* DF is for degrees of freedom.

Considering the effect of the reproductive month, higher ejaculate volumes were obtained at each age in April (Fig. 1) compared with March and May. In May, ejaculate volumes fell to levels lower than those observed in March for almost all ages considered (Fig. 1).

Sperm concentration was greater in April than in March and May (Fig. 2). At the same time, sperm concentration in April decreased along with age, particularly from the 5th to the 7th year of age; thereafter an upswing and slightly higher and constant values were observed. Sperm concentration from samples collected in May were similar to those of March, showing a slightly increasing trend by age (Fig. 2).

Regarding sperm motility (Fig. 3), a higher proportion of motile sperm in April and May than in March was observed at each age, except for the 6 year old-age. From this age on, in March, a progressive worsening throughout aging in sperm motility was observed.

Among the considered semen traits, only sperm viability showed similar values throughout the reproductive season and across ages (Fig. 4).

**Figure 5:**
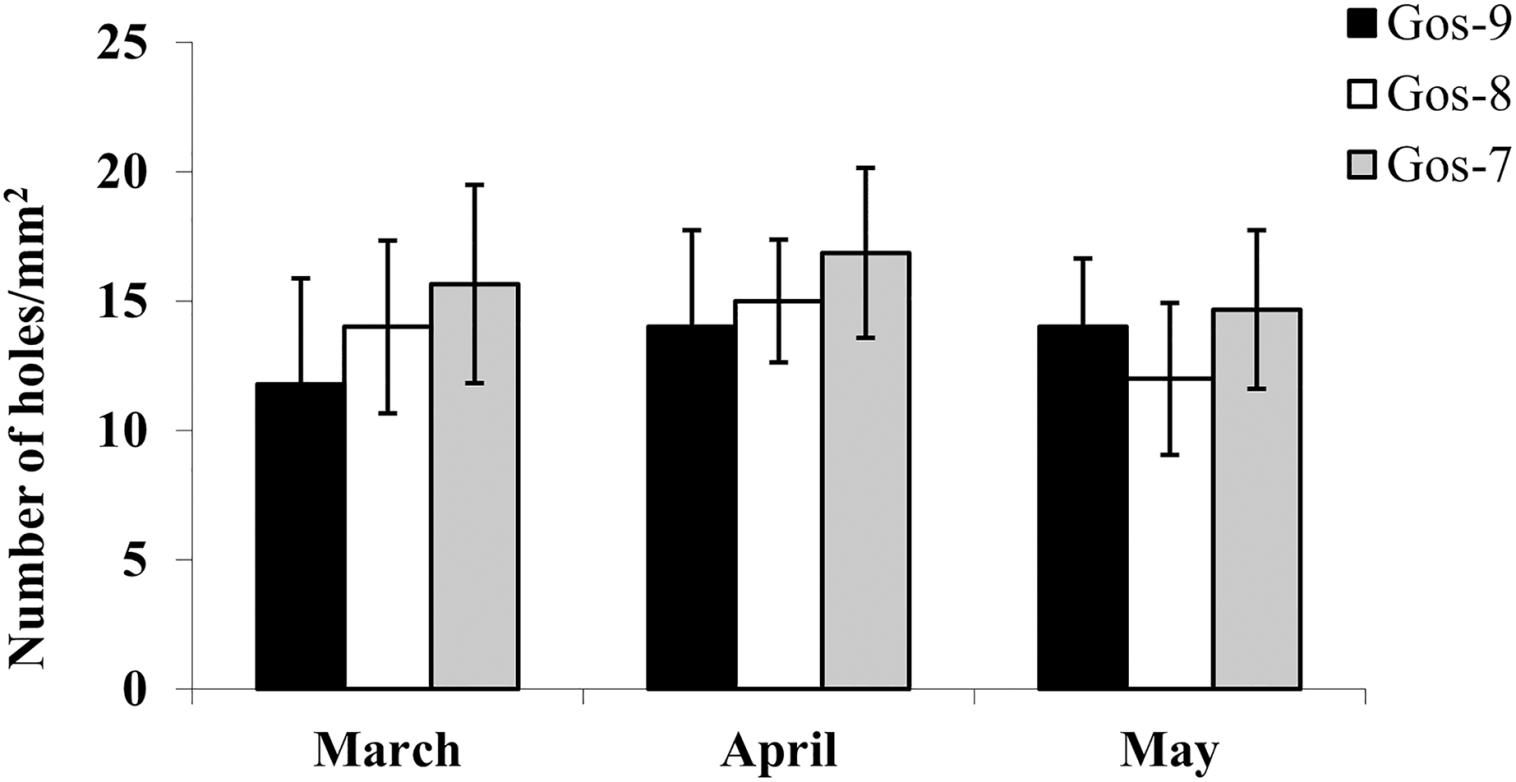
*In vitro* goshawk sperm penetration ability. *In vitro* goshawk sperm penetration ability assessed using the sperm-egg interaction assay on IPL from chicken eggs. The test was performed only on the last year of essay for each individual (Gos-7 = 7-year-old goshawk, Gos-8 = 8-year-old goshawk, Gos-9 = 9-year-old goshawk).

The hydrolytic activity of goshawk spermatozoa towards chicken egg IPL is shown in Fig. 5. The number of holes made by the sperm is visualized per male. In total, the cross reactivity between the goshawk sperm and the heterologous IPL produced 14 ± 3.4 (mean ± standard deviation) hydrolysis points per 1 mm^2^ of IPL.

### Morphological and ultrastructural characteristics of the goshawk sperm cells

#### The normal spermatozoon

**Figure 6:**
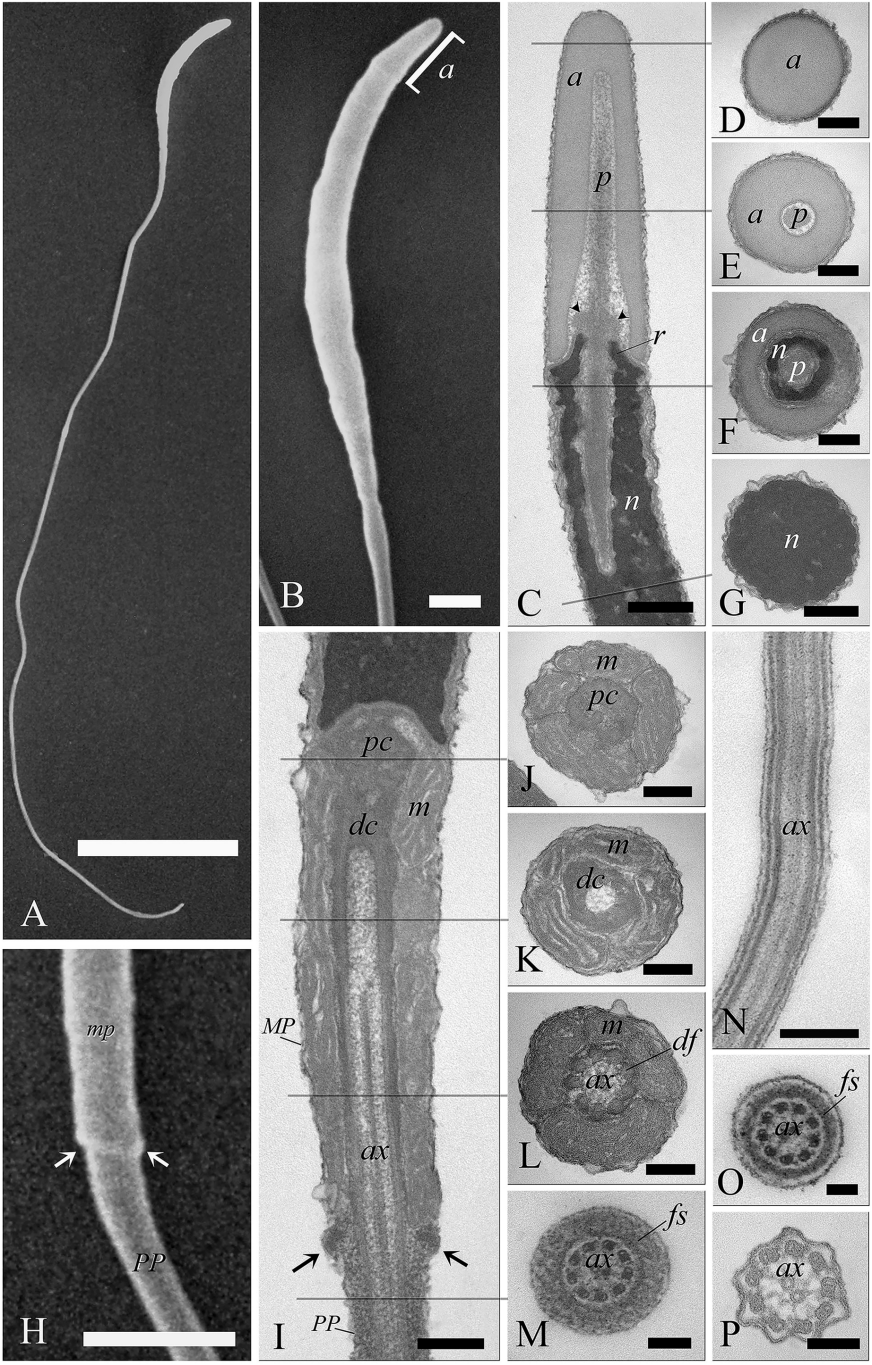
Electron microscopy characterization of the spermatozoon of *A. gentilis*. SEM micrographs (A) of the entire filiform sperm cell, and (B) the head with the acrosome at the anterior tip. (C) Longitudinal sections of the sperm head by TEM. (D–G) Transversal sections at different levels of the sperm head. (H) SEM image of the transition region from the midpiece and the principal piece of the tail. TEM micrographs of respectively the midpiece in longitudinal (I) and cross sections (J–L), and of the principal piece in longitudinal (N) and cross sections (M, O and P). The principal piece of the tail finishes in a short and thinner endpiece (P). Acrosome (a), *annulus* (arrows), axoneme (ax), dense fibers (df), distal centriole (dc), fibrous sheath (fs), midpiece (MP), mitochondria (m), nucleus (n), *perforatorium* (p), principal piece (PP), proximal centriole (pc), and *rostrum* (r). (A) bar: 10 μm. (B) bar: 1 μm. (C–G) bars: 250 nm. (H) bar: 1 μm. (I–L) bars: 250 nm. (M) bar: 100 nm. (N) bar: 250 nm. (O and P) bars: 100 nm.

When viewed by SEM, the spermatozoa were typically filiform in shape, with a total length of 88 ± 1.9 µm (Fig. 6A). The sperm cell is characterized by a slender tapering head (8 ± 0.4 µm in length) capped by a small conical acrosome measuring 1.4 ± 0.3 µm (Fig. 6B). A short midpiece was observed posteriorly to the head (2 ± 0.2 µm), followed by a long tail measuring 78 ± 1.5 µm.

The head of the goshawk spermatozoon was a slightly curved, cylindrical structure (Figs. 6A and 6B). The acrosome formed the anterior tip of the sperm head, and displayed a blunt, rounded tip (Fig. 6B). TEM observations revealed the acrosomal complex to be characterized by the acrosomal vesicle, a cone-like structure composed of homogeneous, moderately electron-dense content (Figs. 6C and 6D), and the *perforatorium*, a rod-like structure lying in the subacrosomal space (Figs. 6C–6E). The *perforatorium* extended proximally to the tip of the sperm cell, and distally inserted within a cylindrical endonuclear canal into the anterior region of the nucleus (Figs. 6C–6F). Focusing on the transition zone from the acrosome to the nucleus, the *perforatorium* showed a thickening at the point where the nucleus was raised to form the so-called nuclear *rostrum* (Fig. 6C). The nucleus was an elongate cylinder structure, characterized by granular, condensed and electron-dense chromatin (Figs. 6C–6G); moreover, the plasmalemma and nuclear membranes were indistinguishable due to the very low amount of cytoplasmic contents. The base of the nucleus terminated in a shallow implantation fossa (Fig. 6I).

The tail was composed of three distinct segments, clearly distinguishable by TEM: the midpiece, the principal piece, and the endpiece. The midpiece of the spermatozoon is characterized by the proximal centriole located in the concave implantation fossa at the base of the nucleus (Fig. 6I). This centriole was positioned on top of, and at right angles to, the distal centriole (Fig. 6I), which together constituted the centriolar complex. In the transverse section, the proximal centriole displayed nine sets of triplet microtubules embedded in a ring of moderately electron dense material (Fig. 6J). The distal centriole, which represented the basal body of the axoneme, was characterized by sparse granular material in its center (Figs. 6I–6K). In longitudinal sections, five to six mitochondria bordered the midpiece (Fig. 6I), while in the transverse section they ranged from 3 to 4 in number (Figs. 6J–6L), with an approximate total of 20–24 mitochondria forming the mitochondrial sheath. The axoneme in the midpiece had nine dense fibers, clearly distinguishable thanks to their high electron density (Fig. 6L), and which did not extend into the principal piece of the tail (Figs. 6M–6P). The midpiece terminated at the *annulus*, a ring of moderately electron-dense material that demarcated the boundary between the midpiece and the principal piece of the flagellum (Figs. 6H and 6I). The principal piece started at the *annulus* and represented the longest part of the tail. This was thinner than the midpiece and was defined by the presence of a fibrous sheath encircling the axoneme (Figs. 6M–6O). This latter became thinner distally and the tail terminated in a short, thinner endpiece that consisted of the axoneme and plasma membrane, lacking the fibrous sheath and characterized by a tapered end (Fig. 6P). The axoneme was composed of nine regularly spaced outer doublet microtubules surrounding a central pair of single microtubules (Figs. 6L–6P).

#### Electron microscopy of sperm defects

**Figure 7:**
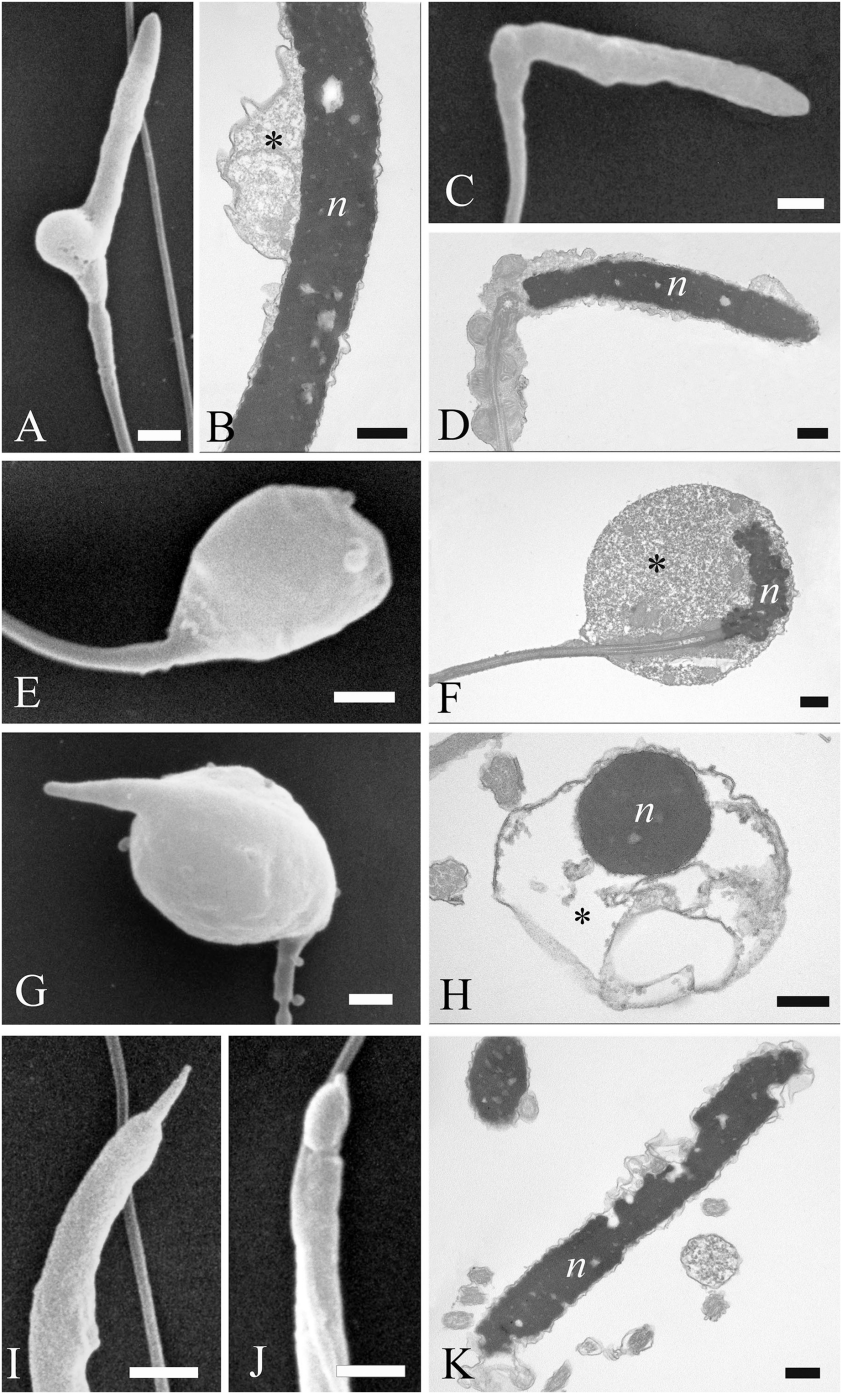
Electron microscopy images of sperm head defects. SEM and TEM images (A and B) of cytoplasmic droplets associated with a weak head bending. (C and D) Morphology and ultrastructure of a severe bending of the head at the proximal region of the midpiece. (E–H) Spermatozoa characterization by SEM and TEM of round heads with a spherical shape. (I and J) Sperm abnormalities on the acrosome shown by SEM, indicating the complete absence of and a swollen acrosomal cap, respectively. (K) TEM image of a nuclear defect, with indentations of the chromatin and the absence of the plasma membrane. Cytoplasmic droplet (*), nucleus (n). (A) bar: 1 µm. (B) bar: 250 nm. (C) bar: 1 µm. (D) bar: 250 nm. (E) bar: 1 µm. (F) bar: 250 nm. (G) bar: 1 µm. (H) bar: 250 nm. (I) bar: 1 µm. (J) bar: 1 µm. (K) bar: 250 nm.

**Figure 8:**
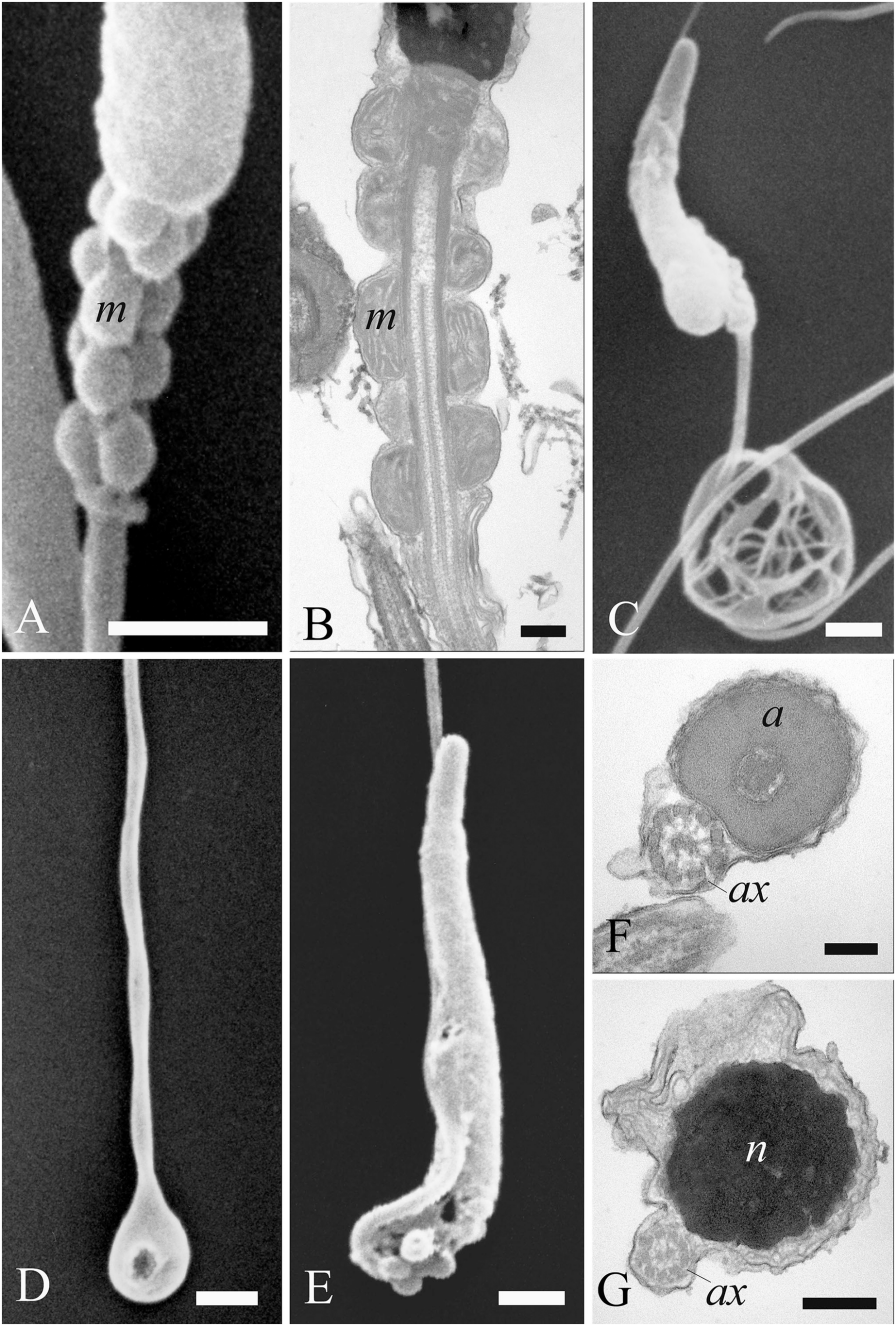
Electron microscopy images of sperm tail defects. SEM and TEM micrographs (A and B) of abnormalities showing the midpiece with a breakage or the absence of the plasma membrane which revealed the individual mitochondria. (C) Abnormalities involving the principal piece of the tail with coiling. (D) Tail forming a loop at the point of folding. (E–G) SEM and TEM images of a tail wrapping around the head, and the plasma membrane covering both structures, as evidenced in cross sections at various levels of the sperm. Acrosome (a), axoneme (ax), mitochondria (m) and nucleus (n). (A) bar: 1 μm. (B) bar: 250 nm. (C–E) bars: 1 μm. (F and G) bars: 250 nm.

A variety of unusual spermatozoa were found in fresh ejaculates of *A. gentilis*. The observations by SEM and TEM allowed us to identify the ultrastructural features of specific abnormalities present in the different regions of the sperm cell (Figs. 7 and 8).

Most abnormalities occurred in the head region, comprising bending, round heads, acrosomal and nuclear defects. Different forms of head bending were identified, ranging from gentle to acute bends. Head bending was often associated with the presence of cytoplasmic droplets of different sizes and located in various positions. Cytoplasmic droplets, which determined the direction of the bend, were often in eccentrical position. They were often located at the base of the head (Figs. 7A and 7B), or at the proximal region of the midpiece (Figs. 7C and 7D), and they were characterized by fine, granular content. When the cytoplasmic droplets involved a larger portion of the head as well as the proximal region of the midpiece, spermatozoa were characterized by spherical heads which varied in size (Figs. 7E and 7F). In some cases, a projection resembling the acrosome was present on round headed sperm, and TEM showed membranous structures present at the swelling (Figs. 7G and 7H). Some sperm completely missed the acrosome cap, revealing the *perforatorium* at the apical region of the head (Fig. 7I). Some rare spermatozoa had a swollen acrosomal cap (Fig. 7J). Ultrastructural analysis by TEM also revealed abnormalities occurring in the nucleus, such as indentations in the chromatin and the absence of the plasma membrane (Fig. 7K).

Concerning the tail, some defects were observed in the midpiece, such as breakages or the absence of the plasma membrane which revealed the individual mitochondria (Figs. 8A and 8B). Other abnormalities involved the coiling of the principal piece of the tail (Fig. 8C) as well as other forms of folding (Figs. 8D and 8G). In one example, the tail formed a loop at the point of folding (Fig. 8D). In another, the tail wrapped around the head (Fig. 8E), with both structures covered by the plasma membrane, as shown in cross sections at various levels of the spermatozoa (Figs. 8F and 8G). Cytoplasmic droplets were never seen in the tail. A small percentage of sperm displayed more than one abnormality simultaneously. Multiple defects mostly combine the presence of cytoplasmic droplets together with bending of the head, or the absence of the plasma membrane in the midpiece together with the lack of the acrosome.

Analysis of the SEM data indicated that about 80.5% of spermatozoa obtained from all three goshawks and during the whole third reproductive season displayed normal morphological features (Table 2). No significant differences were observed between the 3 months in relation to the percentage of normal cells or the percentage of abnormal sperm with head or tail defects. Head abnormalities constituted 8.1% of all defects, whereas tail defects and cytoplasmic droplets were about 3.6% and 2.2% respectively. Multiple defects (about 5.6%) were mostly characterized by cytoplasmic droplets associated with head bending.

**Table 2:** Quantitative SEM analysis of sperm cell morphological characteristics across the 3 months of the third reproductive season (mean ± SD).

	Normal sperm %	Head defect %	Tail defect %	Cytoplasmic droplets %	Multiple %
March	79.3 ± 3.8	8.4 ± 1.2	3.9 ± 1.5	2.5 ± 0.3	5.9 ± 1.2
April	82.3 ± 2.1	7.7 ± 0.8	3.2 ± 0.9	1.8 ± 0.1	5.0 ± 1.0
May	80.0 ± 1.0	8.2 ± 1.1	3.6 ± 1.0	2.4 ± 0.2	5.8 ± 1.1
Total	80.5 ± 3.5	8.1 ± 1.0	3.6 ± 0.5	2.2 ± 0.2	5.6 ± 0.3

#### Artificial insemination and new-born data

All the three imprinted females responded to their handler by assuming copulation postures, everting their cloaca and exposing the oviduct orifice. A total of 24 artificial inseminations were performed using fresh semen, and 19 eggs were obtained, corresponding to an AI success rate of 80% (Table 3). After 15 days of natural incubation, candling confirmed all eggs to be fertilized. Eighteen chicks (six per female) hatched after 34 days of brooding, resulting in a hatching rate of 95%.

**Table 3:** Data on artificial insemination of the three goshawk females, production of fertile eggs and new-borns. The test was performed only on the last year of assay for each individual.

Male × female combination*	Artificial inseminations, n.	Successful event, % (eggs laid, n.)	Egg fertility, %	Hatchability, % (new-borns, n.)
Oldest	9	77.8 (7)	100	85.7 (6)
Intermediate age	8	75.0 (6)	100	100 (6)
Youngest	7	85.7 (6)	100	100 (6)
Total	24	79.2 (19)	100	94.7 (18)

**Note:**

* Animals were combined according to age: the oldest female (10 y.o.) was inseminated with semen from the oldest male (9 y.o.), the 8 y.o. female with semen from the same age male and the youngest female (7 y.o.) with semen from the youngest male (7 y.o).

Regarding the new-born data (10 males and eight females), the average weights at hatch were 36.5 ± 2.7 g and 36.6 ± 2.8 g for male and female pulli, respectively. Ten days after hatch, the new-born survival rate dropped to 80% due to the death of three pulli (1 female and 2 males) characterized by the lowest birth weights. On day 10, the average weight of male pulli was 372.3 ± 42.8 g, whereas that of females was 390.5 ± 49.6 g. At the end of the first month of life, the young goshawks had a mean body weight of 791.6 ± 37.9 g and 1,255 ± 131.9 g in males and females, respectively. The average weight observed in new-borns after 1 month was comparable to that of the adult animals used in this study.

## Discussion

In the present study, goshawk semen obtained by the cooperative method was analyzed to evaluate semen quality for effectiveness in terms of semen traits and assisted reproduction outcome.

In accordance with previous studies on raptors (Temple, 1972; Villaverde-Morcillo et al., 2015), the high percentage of clean ejaculates obtained herein (80%) demonstrates that spontaneous ejaculation induced by cooperative copulation permits a high collection rate of uncontaminated semen, essential for viable and motile spermatozoa (Penfold et al., 2001; Blanco et al., 2002). On the downside, the cooperative method requires a long training period, and in some cases, it is difficult to apply or not convenient as for example in wild birds kept with the goal of wild release. Indeed, the training period could influence their natural habits and survival ability (Dogliero et al., 2016). By considering that minimum semen quality values are required to fertilize the egg (Blanco et al., 2002; Łukaszewicz, Kowalczyk & Rzońca, 2015), several techniques for collecting semen from non-imprinted birds have been developed and are currently under consideration, *e.g.*, the massage method, the cloaca flushing or the cloaca electrostimulation. However, some of these techniques cannot guarantee the high success rate achieved by the cooperative method because these are stressful to the birds and involve the capture and manipulation of non-sedated birds. The influence of the semen collection method on the quality of the fresh semen has been clearly demonstrated in a number of avian species (Castillo, Marzoni & Romboli, 2009; Łukaszewicz, Kowalczyk & Rzońca, 2011; Castillo et al., 2021; Tang et al., 2021). The massage method, the most common approach for semen collection (Łukaszewicz, Kowalczyk & Rzońca, 2015; Dogliero et al., 2016; Piacek et al., 2020), may also stimulate urination and/or blood emission in the case that the handler’s dexterity is insufficient, with the consequent contamination of the ejaculate (Madeddu et al., 2009). It is known that a watery ejaculate or its contamination with faeces markedly reduces sperm longevity and their fertilizing capacity (Lake, 1971; Fujihara & Nishiyama, 1976). The electrical stimulation method, although proved feasible, is very time-consuming because it requires deep sedation or anesthesia of the animal and has the potential to cause damage to internal tissues (Frediani et al., 2019).

High-quality ejaculates are essential for improving the outcome of assisted reproduction techniques for the propagation of captive rare avian species. The analysis of semen characteristics prior to AI are also important information for identifying the best-performing donors and best-quality semen (Łukaszewicz & Kowalczyk, 2015; Krohn et al., 2019; Cardoso et al., 2020; Castillo et al., 2021).

With respect to the month of the reproductive season, ejaculate volume and sperm concentration showed similar trends, with maximum values recorded in April regardless of the age, thus revealing the peak of the male goshawk reproductive season in April. Likewise, cell motility was greatest in April, but similar quality was evident in the following month as well, suggesting that the species can produce high-performing cells at the end of the reproductive season too. Additionally, sperm motility was negatively correlated to age only at the beginning of the reproductive season.

Finally, our study showed that a high viability of sperm cells was maintained independently from age and the time within the reproductive season. According to our data, the results suggest that a variation in the sperm viability may be due to the factors studied (see Table 1), but differences among treatments (combined factors) could not be retrieved after the adjustment of multiple comparisons (see Fig. 4 *vs*. unadjusted *p*-differences for the same variable in the Supplemental Material table “SAS results” from line 1,028 to line 1,135).

The seasonality probably reflects the natural fluxes in testosterone production, which peaks in the middle of breeding season, correlating with the height of the animals’ reproductive period, as previously demonstrated for other raptor species (Blanco, Bird & Samour, 2007).

Considering that this species lives for up to 19 years in the wild and usually reaches sexual maturity at 2 to 3 years of age (del Hoyo, Elliott & Sargatal, 1994; Squires et al., 2020), the age seems not to affect the semen production, as reported for goshawk aged between 2 and 15 years (Reynolds et al., 2019). Our study contrasts with these latter results. Indeed, the total semen production (*i.e.*, the product of volume by concentration) was 11.35 million sperms per ejaculate (on average, data not showed) in individuals aged 5-years old, and such an amount reduced by 22%, 50%, 55% and 70% in individuals aged 6-, 7-, 8-, and 9-years old, respectively. Such variations strongly correlated (r = 0.999) with the mean reduction of the sperm concentration by age.

It is also interesting to point out that the average ejaculate volume (49.1 ± 23.8 µL) was much higher than those reported by Dogliero et al. (2016) for *Accipiter nisus* (2 µL) and other Accipitridae of comparable sizes: *Falco subbuteo* (3.6 ± 2.4 µL), *Falco tinnunculus* (2.8 ± 1.3 µL) and *Bubo bubo* (9.0 ± 4.0 µL). This large difference in semen volume obtained from similarly sized Accipitridae is likely due to the semen collection procedure employed, since Dogliero et al. (2016) adopted the massage method.

On the contrary, the concentration of spermatozoa in ejaculates observed in our study (41.0 ± 25.4 × 10^6^/mL) was lower than that reported by Piacek et al. (2020) for the same species (110.2 ± 63.2 × 10^6^/mL). This difference is probably linked to the duration of the collection period, as the latter authors evaluated semen collected in April only. In our study, the closest value in sperm concentration was observed in April at 5 years of age (85.3 ± 10.8 × 10^6^/mL). Other authors reported also sperm concentration values in raptors such as *Gyps bengalensis* (58.4 × 10^6^/mL) (Umapathy et al., 2005), and the prairie falcon (20.0 × 10^6^/mL) (Boyd, Boyd & Dobler, 1977).

On the basis of our observations, sperm motility (78.5 ± 10.3%, see Supplemental Materials for the number of replicates per sample) was higher than previously reported for the same species (51.6 ± 22.8%; Piacek et al., 2020) and for other birds of prey species, such as *Accipiter nisus* (18%), *Aquila heliaca* (32.1 ± 14.9%), *Falco peregrinus* (41.2 ± 18.7%), *Falco subbuteo* (49.2 ± 37.1%), *Falco tinnunculus* (59.2 ± 27.1%), *Bubo bubo* (25.2 ± 18.0%) and *Gyps bengalensis* (46.8 ± 16.5%) (Umapathy et al., 2005; Dogliero et al., 2016; Piacek et al., 2020). Furthermore, these results could depend on the semen collection method applied to the males. For instance, a comparison of semen collection methods in drakes (male stimulated by the female (induction method) *vs*. massage) found higher sperm motility values in both fresh and frozen semen with the former method (71% and 33% *vs*. 61% and 19%, respectively) (Tang et al., 2021).

The morphology of both normal and abnormal goshawk sperm was analyzed during the last breeding season considered. Sperm morphology is one of the most important qualitative parameters to consider in semen evaluation (du Plessis & Soley, 2014; Støstad et al., 2018). The present work describes, for the first time, the normal ultrastructure of the spermatozoon of *A. gentilis*. In the literature, the only ultrastructural study of Falconiformes sperm consists of a scanning electron micrograph of peregrine falcon spermatozoon (Jamieson, 2011). The morphological features of the sperm cell evaluated in the present study allowed us to classify the goshawk spermatozoa as a non-passerine type, the more “primitive” filiform sperm type, which is characterized by five clearly defined segments: the acrosome and the nucleus forming the head, and the midpiece, the principal piece and the endpiece constituting the tail (Jamieson, 2011; van der Horst, 2021).

In addition to providing information on normal sperm morphology, ultrastructural investigations provide a reference database of morphological categories of sperm defects present in fresh semen. These data can then be referred to when assessing the effects of different sperm storage techniques. The ultrastructural analysis revealed the percentage of abnormal sperm cells in fresh goshawk semen to be low, and similar to the values obtained in other avian species, which should therefore be considered physiological (Penfold et al., 2000; Alkan et al., 2002; Umapathy et al., 2005; Santiago-Moreno et al., 2009; du Plessis & Soley, 2011; Łukaszewicz, Kowalczyk & Rzońca, 2011).

Functional tests are recommended to complement semen assessments, providing information that cannot be obtained from semen motility data or viability rates (Bailey & Lierz, 2017). In this view, we performed both the sperm-egg interaction assay and subjected female goshawks to artificial inseminations.

In the sperm-egg interaction assay, the sperm penetration rate of the chicken perivitelline membrane was assessed, and we showed that the sperm from all three goshawks were able to produce holes in the IPL, and thus likely to fertilize an egg.

In fact, the interaction between spermatozoa and the IPL requires that a series of functions are performed, including motility, binding to the IPL, induction of the acrosome reaction and hydrolysis of the IPL, meaning that the test results are highly discriminatory towards compromised spermatozoa (Krohn et al., 2019). In addition, this method does not require advanced technology and is cheap and simple to perform (Robertson et al., 1997).

Moreover, our results show, for the first time, the ability of the goshawk spermatozoon to perform hydrolytic activity towards the IPL of the chicken egg, a phylogenetically distant species. Stewart et al. (2004) counted a mean of 18 holes/mm^2^ in the chicken egg IPL following the interaction of 12.5 million chicken spermatozoa with the membrane (homologous interaction). In the same study, the cross-reactivity between chicken sperm and the IPL from eggs of some different species (heterologous interaction) demonstrated a decreasing degree of interaction as the phylogenetic distance between species increased; the authors reported low chicken sperm hydrolytic activity towards the IPL from bald eagle (*Haliaeetus leucocephalus*), a species in the Accipitridae family. In the study by Krohn et al. (2019), falcon sperm from the European kestrel (*Falco tinnunculus*) used at the concentration recommended for roosters, did not produce any visible holes in chicken IPL; the authors attributed the failed interaction to use of the chicken egg membrane, thus concluding that chicken IPL cannot be used to assess falcon sperm. By contrast, the present study demonstrated, for the first time, the ability of *A. gentilis* sperm to interact *in vitro* with the IPL from the chicken egg despite the phylogenetic distance between the two species. The high quality of the sperm incubated with the membrane and the slightly higher cell concentration used in our study (1.5–2 × 10^7^ sperm/mL instead of 1.25 × 10^7^ cells/mL) allowed us to confirm the capacity for cross-reactivity and the validity of this assay for evaluating the fertilizing ability of spermatozoa in a phylogenetic distant species. It is important to underline that 1.25 × 10^7^ cells/mL have previously been considered an optimal sperm concentration for homologous interaction in chicken (Robertson et al., 1997). Moreover, others confirm the appearance of visible holes, albeit very few, when applying this sperm concentration for species that are phylogenetically very distant from the Galliformes family, such as those of the Accipitridae family (Stewart et al., 2004). Our findings suggest for heterologous interactions the necessity for high quality cells and a higher sperm concentration, in order to achieve successful hydrolytic activity and to obtain visible holes.

The present study evaluated the outcome of artificial inseminations in the goshawk, using the cooperative method and involving three different male-female age combinations. Eighty percent of the attempts were successful, eggs hatched in 95% of cases, and the new-born survival rate, 10 days after hatch, stood at 80%. These results are comparable to those obtained in domestic species, where the percentage of eggs fertilized by artificial insemination ranges between 85% and 95% (Joyner, 1994). Healthy, naturally reproducing avian populations generally show a fertility rate of 80% or higher (Piacek et al., 2020). Moreover, the equal number of births obtained from the three mating couples is consistent with our laboratory observations on semen traits, demonstrating the reproductive validity of spermatozoa in ensuring a complete embryonic development when adult goshawks reach the 7th to 10th year of age. It is known that the number of sperms needed for fertilization is not the same as the number of sperms needed for normal embryo development. Although minimal quality values must be attained to fertilize the egg (Blanco et al., 2002; Łukaszewicz, Kowalczyk & Rzońca, 2015; Dogliero et al., 2016), low sperm numbers are also associated with early embryo mortality, and supernumerary sperm is key factor in early embryogenesis, improving the likelihood of normal embryo development (Hemmings & Birkhead, 2015; Mizushima, 2017).

## Conclusions

The present study indicates that spontaneous ejaculations resulting from cooperative copulation permits the collection of highly promising semen, especially when compared with values reported in bibliography that are obtained by other methods. Although the cooperative method is challenging as it is very expensive and, in some cases, it is difficult or not convenient to apply, it may be recommended for reproductive biotechnology, such as AI and semen cryopreservation, where high sperm quality is required. Furthermore, the provided information can be useful for workers with non-imprinted animals with a benchmark to assess the impact of sampling stress on sperm characteristics.

The quali-quantitative evaluation of the semen provided useful data on the characterization of semen traits in relation to seasonality.

Most of semen samples collected from 5 to 9 years old males were considered to be of good quality. In the central month of the reproductive season, the bird age did not influence male goshawk performance in terms of fertilization potential of the sperm from 7 to 9 years old goshawk, as we demonstrated by both the *in vitro* (perivitelline membrane assay) and *in vivo* (artificial inseminations) functional analyses.

The results of this study provide a basis for choosing semen donors and for identifying the best semen-sampling period for AI technology in captive *A. gentilis*. Further studies are needed to develop suitable protocols for the purpose of semen cryopreservation. The findings from this study will facilitate similar investigations into other species, in particular those at risk of extinction.

## Supplemental Information

10.7717/peerj.15094/supp-1 Supplemental Information 1 SAS results, Table fixed effects, means, sd and counts, and raw data. All data related to the semen parameters analysed for the three males (A, B and C) during the three consecutive reproductive seasons (2012–2014): volume, sperm concentration, sperm motility, live sperms and the rate of motile/immotile and live/death sperms.

## Additional Information and Declarations

### Data Availability

The following information was supplied regarding data availability: The raw data is available in the Supplemental File.
